# Reduced monoaminergic nuclei MRI signal detectable in pre-symptomatic older adults with future memory decline

**DOI:** 10.1038/s41598-020-71368-1

**Published:** 2020-10-30

**Authors:** Annalena Venneri, Matteo De Marco

**Affiliations:** Department of Neuroscience, University of Sheffield, Beech Hill Road, Sheffield, S10 2RX UK

**Keywords:** Neuroscience, Psychology, Medical research, Neurology

## Abstract

Evidence from murine models and human post-mortem studies indicates that monoaminergic nuclei undergo degeneration at the pre-symptomatic stage of Alzheimer’s disease (AD). Analysing 129 datasets from the Alzheimer’s Disease Neuroimaging Initiative (ADNI) and relying on the Clinical Dementia Rating as group-defining instrument, we hypothesised that the MRI signal of monoaminergic nuclei would be a statistically significant predictor of memory decline in participants initially recruited in ADNI as healthy adults. As opposed to a group of cognitively stable participants, participants developing memory decline had reduced signal in the ventral tegmental area at baseline, before any evidence of functional decline emerged. These findings indicate that monoaminergic degeneration predates the onset of memory decline in an AD-centred initiative, with a crucial involvement of very-early changes of a dopaminergic region. This translates into potential informative avenues for pharmacological treatment of pre-symptomatic AD.

## Introduction

Cerebral accumulation of beta amyloid and neurofibrillary tangles are widely regarded as central pathophysiological processes at the earliest preclinical stages of Alzheimer’s disease (AD)^1^. A prominent body of studies, however, has shown that other neural changes predate the onset of amyloidosis and TAU hyperphosphorylation. These changes affect some of the small monoaminergic nuclei located in the brainstem: locus coeruleus (LC)^2,3^, dorsal raphe (DR)^4^, and ventral tegmental area (VTA)^5,6^.

The LC is the first region of the brain showing non-neurofibrillary TAU-related changes before any cortical deposition of beta amyloid or tangle pathology^2^. The neurons of this nucleus “*accumulate aberrant tau species for decades before frank LC cell body degeneration in AD*”^3, page 1]^. The transition from Braak Stage 0 to 1 corresponds to a ≈ 8% volume loss in the LC^7^. Structural MRI can capture this volumetric change with a decrease in T1-weighted (T1W) image signal^8^. Similar to LC, hyperphosphorylated TAU-related changes are observed in the DR prior to any tangle deposition detected in the transentorhinal region^4^. No experimental study, however, has yet provided evidence of a significant structural or functional change in this nucleus at the preclinical stage of AD. At present, this link has only been hypothesised^9^.

It has been known for several decades that neurofibrillary pathology in the mediotemporal lobe arises following lesions of the VTA, raising the possibility that dopaminergic denervation is a cause of tangle formation^5^. Consistently with this hypothesis, a study run in the Tg2576 mouse model of AD found significant neuronal loss in the VTA at a stage when no Aβ or TAU pathology was yet visible^6^. Importantly, apoptosis of dopaminergic neurons was correlated with a trend of cellular depletion in the hippocampus and reduction in memory performance^6^. In a follow-up study, it was found that the size of the VTA is correlated with hippocampal volume and memory performance in humans as well^10^. These studies indicate that neural properties of LC, DR and VTA may play an informative role in the pre-symptomatic phase of AD and can thus predict the future development of memory decline. On these bases, we formulated an experimental hypothesis to be tested in a cohort of cognitively unimpaired participants recruited as part of an AD-centred initiative and followed up clinically over multiple timepoints. Specifically, we focused on the concept of functional memory decline as assessed by the Clinical Dementia Rating (CDR) scale^11^, and we hypothesised that the MRI signal extracted from the three aforementioned monoaminergic nuclei would show significant cross-sectional and longitudinal differences between declining and stable participants.

## Methods

### Participants

Data used in the preparation of this article were obtained from the Alzheimer’s Disease Neuroimaging Initiative (ADNI) database (adni.loni.usc.edu). The ADNI was launched in 2003 as a public–private partnership, led by Principal Investigator Michael W. Weiner, MD. The primary goal of ADNI has been to test whether serial MRI, positron emission tomography, other biological markers, and clinical and neuropsychological assessment can be combined to measure the progression of mild cognitive impairment (MCI) and early AD. All ADNI participants provided written informed consent, and study protocols were approved by each participating site’s institutional review board. All methods were carried out in accordance with the relevant guidelines and regulations. For research governance and compliance with ethical standards and informed consent please consult the ADNI website at www.adni-info.org and associated material. Additional local ethical approval was not required since the ADNI database contains only anonymised data that are publicly available for download.

The ADNI database was consulted to identify participants who had been recruited as healthy adults with no cognitive impairment and later developed functional memory decline at one of the subsequent follow ups. Longitudinal progression on the global score of the CDR scale was used to operationalise decline. The CDR is a 5-score scale developed by the University of Washington (USA) and used to stage AD and other types of dementia^11^. The global CDR score (CDR-GS) is a valid measure to detect longitudinal functional decline^12^. In this study, functional decline was defined as a CDR-GS increasing from 0 to  0.5 at one of the follow ups. Based on the standard scoring algorithm, memory is the most important CDR category, and is central to CDR-GS scoring. For this reason, functional memory decline is the main drive of a CDR-GS increase from 0 to  0.5. A memory CDR = 0 corresponds to "*no memory loss or slight inconsistent forgetfulness*" while progression to 0.5 tracks the onset of "*consistent slight forgetfulness; partial recollection of events; ‘benign’ forgetfulness*”.

**Figure 1:**
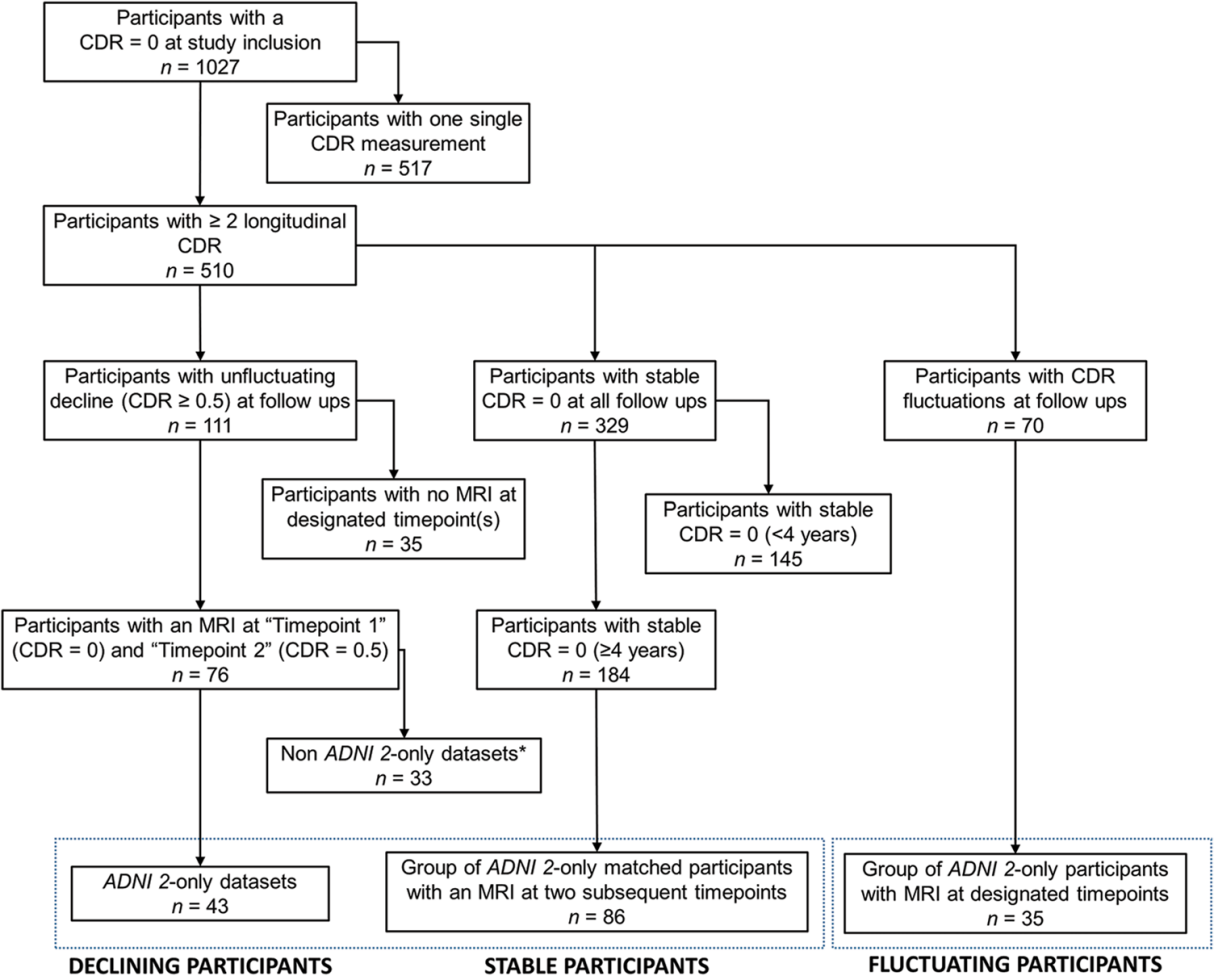
Flowchart showing the procedure of sample selection from the ADNI database. The dotted frames identify the three groups of participants included in the study.

To rule out the effect of simple fluctuations, individual follow-up profiles were inspected to draw a precise CDR-GS trend for each participant. Participants showing a fluctuating trend (i.e., CDR-GS = 0 increasing to 0.5 and then shifting back to 0) were not included in the main study, but were analysed as a separate group. The outcome of this selection was a sample of participants with no initial deficits progressing to functional memory decline devoid of any documented fluctuations (*n* = 76). Two timepoints were defined for each of these participants: a “Timepoint-1” representing the last CDR-GS = 0 measurement with an available MRI image before conversion to a CD-GS = 0.5 and a “Timepoint-2” representing the first CDR-GS = 0.5 assessment with an available MRI image. Since the entire ADNI enterprise is split in four separate sub-initiatives that differ from one another in their protocol of MRI acquisition and in scanner field strength, only datasets acquired entirely (i.e., at both timepoints) as part of ADNI-2 were retained (*n* = 43). An ADNI-2 sample of 86 healthy research participants with CDR-GS = 0 stable for at least 4 years was selected as control group (Fig. 1). As part of the procedures carried out by ADNI, all participants provided their informed written consent.

### MRI specifications and processing

Three-dimensional T1W MRI images were extracted for each participant. These included two images acquired in correspondence with Timepoint-1 (CDR-GS = 0 for all participants) and Timepoint-2 (CDR-GS = 0.5 for declining participants; CDR-GS = 0 for stable participants). The specifications of ADNI-2 T1W scans were as follows: 3 T magnetic field, 8-channel head coil, repetition time 400 ms, flip angle 11°, resolution 256 × 256, field of view 26 cm and slice thickness 1.2 mm). Each image was carefully inspected to rule out signal artefacts. The characteristics of the final sample are illustrated in Table 1.

**Table 1:** Demographic and global neurostructural indices of the two groups of participants.

Variable	Stable participants (*n* = 86)	Declining participants (*n* = 43)	Sig. *t* test
*Demographic indices*			
Age (years)	75.16 (4.75)	78.15 (6.15)	0.003
Education (years)	16.43 (2.63)	15.93 (2.62)	0.310
Gender (f/m)	49/37	25/18	0.900
APOE genotype (ε2ε2/ε2ε3/ε2ε4/ε3ε3/ε4ε3/ε4ε4)	0/8/0/51/24/3	0/2/1/27/12/1	0.561
Timepoints-1-2 distance (days)	480.07 (201.16)	491.02 (252.29)	0.805
Total intracranial volume (ml)	1487.20 (149.81)	1491.61 (157.68)	0.877
*Cerebrospinal fluid AD biomarkers*			
Beta Amyloid_1-42_ (pg/ml)	1412.81 (600.21)	1049.72 (616.56)	0.011
Total TAU (pg/ml)	238.60 (87.05)	271.69 (126.96)	0.242
Phosphorylated TAU (pg/ml)	21.80 (8.70)	26.20 (14.20)	0.161
*Global neurostructural indices – Timepoint-1*			
Grey matter volume (ml)	622.55 (62.25)	583.17 (74.68)	0.002
White matter volume (ml)	405.75 (54.57)	386.77 (59.21)	0.073
Left hippocampal volume (ml)	2.61 (0.31)	2.37 (0.40)	< 0.001
Right hippocampal volume (ml)	2.69 (0.32)	2.44 (0.43)	< 0.001
*Global neurostructural indices – Timepoint-2*			
Grey matter volume (ml)	612.56 (62.78)	569.82 (74.62)	< 0.001
White matter volume (ml)	402.85 (53.86)	384.93 (59.34)	0.088
Left hippocampal volume (ml)	2.58 (0.31)	2.31 (0.38)	< 0.001
Right hippocampal volume (ml)	2.65 (0.33)	2.38 (0.43)	< 0.001

Cerebrospinal fluid analyses were run on 103 out of 129 participants.

All MRI images were processed using Matlab (Mathworks Inc., UK) and Statistical Parametric Mapping 12 (Wellcome Centre for Human Neuroimaging, London, UK). Each image was segmented to extract the map of grey matter that was then smoothed with an 8 mm full-width at half maximum Gaussian kernel. By doing so, the numerical information associated with each voxel represented the amount of grey matter found in the region encircling the voxel^13^. Additionally, global volumes of grey matter, white matter, and cerebrospinal fluid were quantified using the “get_totals” command line (https://www0.cs.ucl.ac.uk/staff/g.ridgway/vbm/get_totals.m) in Matlab for the calculation of total intracranial volumes.

### AD biomarkers

Cerebrospinal fluid biomarkers were available from ADNI for 103 of 124 participants (24 and 79 from the groups of declining and stable participants, respectively) at a temporal distance of < 1 year from Timepoint-1 MRI. Hippocampal volumes were also extracted. These were calculated with an automated procedure that makes use of multiple templates^14^, and available at https://niftyweb.cs.ucl.ac.uk/.

### Signal extraction from monoaminergic nuclei

Given the small size of the monoaminergic structures of interest, particular care was taken to define a valid and reliable procedure for the extraction, processing and analysis of the T1W-based signal from each nucleus. A binary mask was created for each monoaminergic nucleus of interest (LC, DR and VTA) and for two control regions, i.e., the substantia nigra and the nucleus basalis of Meynert. The definition of all five nuclei was carried out with a methodology informed by the procedures detailed in a number of peer-reviewed publications: DR signal was extracted from a 32 mm^3^ ROI centred at x = 0, y = − 27, z = − 9, Montreal Neurological Institute coordinates, as described in previous studies^15–17^. The probabilistic template, calculated by Keren and colleagues^18^, was used to define the LC as done in previous research^19,20^. The VTA was drawn as a sphere of 3-mm radius centred at x = 0, y = − 16, z = − 7 following the procedure implemented in previous studies^10,21,22^.

**Figure 2:**
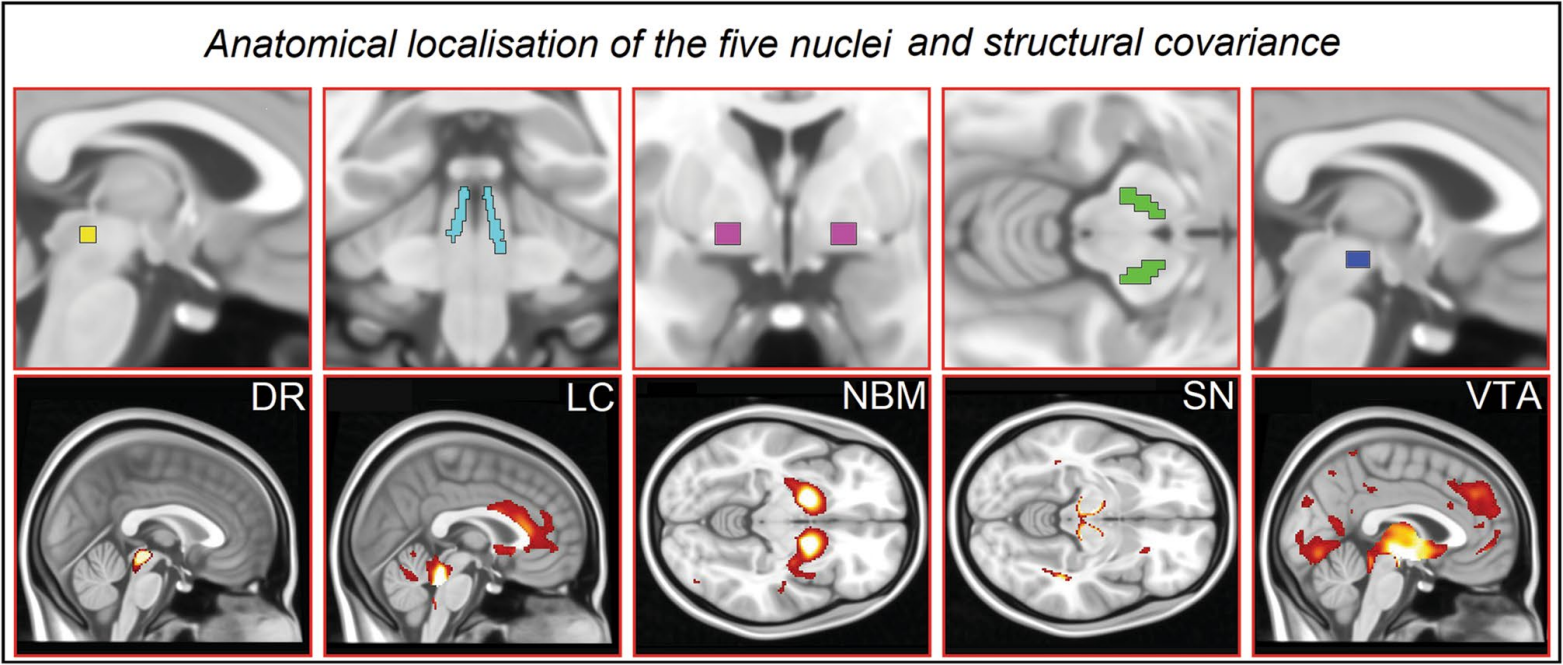
Subcortical nuclei investigated in this study and their structural covariance (in alphabetical order), superimposed to the standard Montreal Neurological Institute space.

The substantia nigra was selected as a monoaminergic control region. In fact, this nucleus is mostly susceptible to aggregation of alpha-synuclein and linked to the presence of motor rather than cognitive symptoms^23^. This nucleus was defined from the Brodmann atlas available on the WFU Pickatlas toolbox^24^, following the procedure successfully validated by previous publications^25–27^. A second, non-monoaminergic nucleus was also included in the analyses as further methodological control. The nucleus basalis of Meynert was defined based on the probabilistic mapping of magnocellular compartments of the basal forebrain^28^. Two spherical masks (3-mm radius each, central coordinates in the Montreal Neurological Institute space: x =  ± 16, y = 0, z = − 8) were superimposed to the Ch4 cell group, the subcommissural portion of the basal forebrain that has been historically identified as the nucleus basalis of Meynert^29,30^. All five regions are illustrated in Fig. 2.

**Figure 3:**
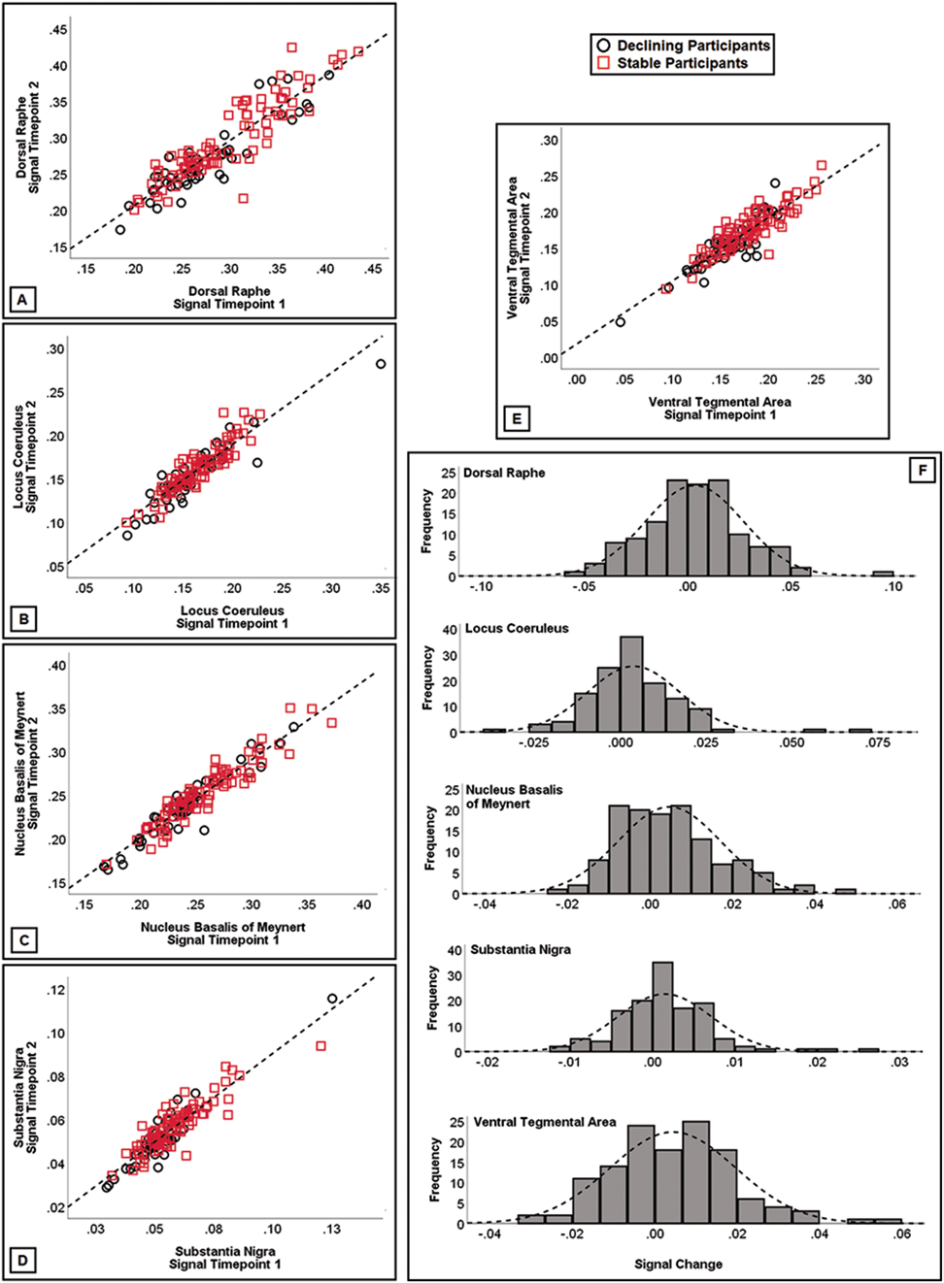
Signal consistency within each nucleus (**A**–**E**) between Timepoint-1 and Timepoint-2. All associations were significant (*r* > 0.89), supporting test–retest reliability of T1-weighted signal from these small regions. A signal change index was calculated subtracting the signal at Timepoint-2 from the signal at Timepoint-1. This index was plotted (F) to confirm normality of data for longitudinal analyses.

**Figure 4:**
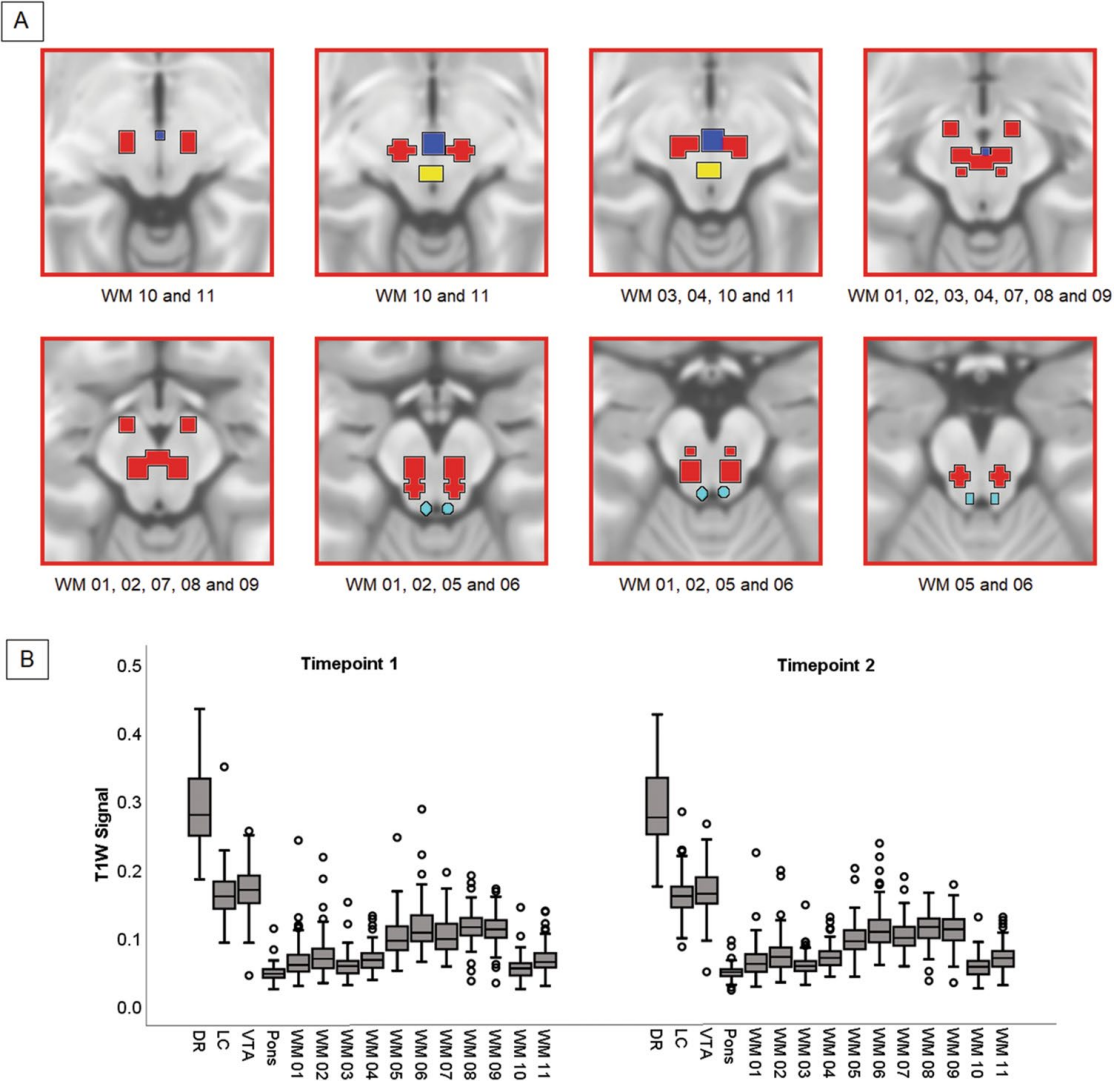
Definition of pontine white matter regions for extraction of T1-weighted signal. (**A**) Eleven spherical regions (3-mm radius) were created around the following Montreal Neurological Institute coordinates. WM 01: x = − 6, y = − 24, z = − 15; WM 02: x = 6, y = − 24, z = − 15; WM 03: x = − 6, y = − 19, z = − 11; WM 04: x = 6, y = − 19, z = − 11; WM 05: x = − 6, y = − 30, z = − 18; WM 06: x = 6, y = − 30, z = − 18; WM 07: x = 0, y = − 21, z = − 13; WM 08: x = − 9, y = − 11, z = − 13; WM 09: x = 9, y = − 11, z = − 13; WM 10: x = − 9, y = − 20, z = − 8; WM 11: x = 9, y = − 20, z = − 8. These regions were created to keep the overlap to a minimum and cover a large portion of the pons. The DR, LC and VTA are indicated in yellow, light blue and blue, respectively. z slices in the Montreal Neurological Institute space are: − 6, − 8. − 10, − 12, − 14, − 16, − 18, − 20. The white-matter regions shown in each slice are indicated below each image. (**B**) The graphical representation of the T1-weighted signal from the three monoaminergic nuclei and the eleven white-matter regions (plus the global pons), showing significant differences in intensity.

The T1W signal intensity was extracted from the binary topography of each nucleus superimposed to the set of smoothed grey matter maps. Additionally, the signal from the pons was also extracted from all scans as correction factor to be used in cross-sectional models. To test for test–retest reliability of signal extracted from each nucleus, a coefficient of correlation was calculated to test the association between the signal calculated at Timepoint-1 and Timepoint-2. These linear associations, shown in Fig. 3, were extremely strong (all *r*-coefficients > 0.89). Vice versa, coefficients of correlation calculated between nuclei (e.g., DR at Timepoint-1 and VTA at Timepoint-2) were considerably smaller (*r* = 0.5, on average). In addition, the map of structural covariance was calculated for each nucleus, to confirm the appropriateness of the extracted signal (Fig. 2). Furthermore, the signal from 11 pontine regions of comparable size as the VTA (spheres with a 3-mm radius), but fully enclosed within the segmented white-matter map, was also extracted. This served to assess the magnitude of the signal of the experimental nuclei of interest and compare it to the signal of areas devoid of grey matter. The localisation of these areas and the graphical representation of their signal are illustrated in Fig. 4. The signal from the experimental nuclei of interest was significantly stronger compared to these white-matter regions. This strongly supports the genuineness of the measurements and minimises the possibility that the signal included in the analyses was of artefactual nature.

### Statistical analysis

#### Signal from the nuclei

Data analysis was run using ISBM SPSS Statistics 23 (https://www.ibm.com/uk-en/analytics/spss-statistics-software). Normality of dependent variables was tested with the *Kolmogorov–Smirnov Goodness-of-fit test*. Since the study was based on cross-sectional as well as longitudinal designs, normality of signal residuals was tested at each timepoint and on a timepoint-to-timepoint subtractive signal change index (Fig. 3f). No breach of the assumption was detected.

To test for signal differences at the two timepoints, one-way *ANOVA*s were run comparing the two groups of participants. Age, the signal from the whole pons and the signal from all eleven pontine regions described in “Signal extraction from monoaminergic nuclei” were used as correction factor.

To test for differences in the longitudinal progression of signal between the two groups, mixed *ANOVA*s were run testing the effect of the 2 × 2 group-by-timepoint interaction. The temporal distance between timepoints (in days) was added as covariate to these models. A Bonferroni-corrected *p* < 0.01 (0.05/*n* of tested nuclei) was selected as threshold of significance. Two-tailed hypotheses were tested.

#### MRI whole-brain

Comparable inferential models as those described in the previous paragraph were devised to analyse T1W images voxel-by-voxel following the procedures of voxel-based morphometry^31^. These analyses were conducted using Statistical Parametric Mapping. Two-sample *t*-test models were run to compare the two groups at each timepoint, controlling for signal extracted from the pons and from the entire map of grey matter. For these analyses, a standard set-level uncorrected *p* < 0.005 was selected. Only clusters surviving a cluster-level Family-Wise Error corrected *p* < 0.05 were retained as significant. Montreal Neurological Institute coordinates were converted to the Talairach space via a nonlinear transformation (imaging.mrc-cbu.cam.ac.uk/downloads/MNI2tal/mni2tal-m). Spatial coordinate interpretation was drawn using the Talairach Daemon Client^32^.

#### Association between nuclei signal and AD biomarkers

The association between nuclei signal and cerebrospinal-fluid AD biomarkers (Beta Amyloid_1-42,_ total TAU and phosphorylated TAU) was also tested. Partial correlation models were devised. Age, signal from the pons and temporal distance (in days) between MRI and lumbar puncture were used as controlling factors. A Bonferroni-corrected *p* < 0.01 threshold (0.05/*n* of tested nuclei) was used for these analyses.

#### Analysis of participants with fluctuating CDR

A group of 35 ADNI-2 participants with a Timepoint-1 CDR = 0, a Timepoint-2 CDR = 0.5 and subsequent reversion to CDR = 0 was also investigated in a series of ancillary analyses. A detailed description of this sample and the results of cross-sectional and longitudinal analyses are reported as Supplementary Material.

## Results

The groups of stable and declining participants were matched for education, gender, *APOE* genotype and temporal distance between the two scans, but the group of stable participants was slightly, yet significantly younger and had more grey matter, globally (Table 1). Declining participants had also significantly smaller concentrations of beta amyloid, while there were no differences in TAU levels.

### Signal from the nuclei

**Figure 5:**
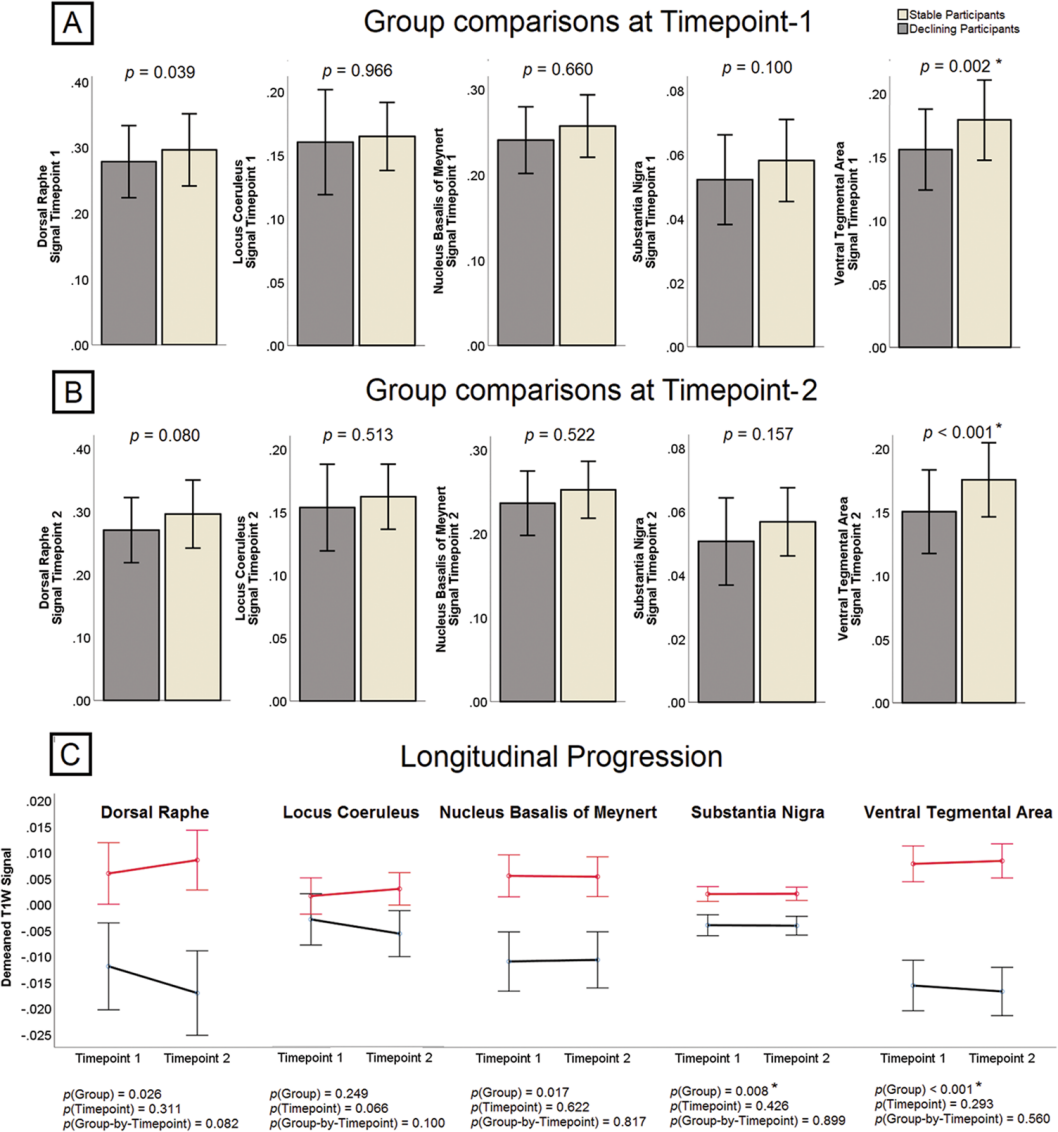
(**A**, **B**) Between-sample comparisons between the group of stable (*n* = 86) and declining (*n* = 43) participants at the two timepoints. Graphs indicate arithmetical means and error bars represent one standard deviation. (**C**) Graphical representation of the mixed *ANOVA* models. Black and red lines show declining and stable participants, and error bars indicate the standard error of the mean. *p < 0.01 (Bonferroni-corrected threshold of significance).

**Figure 6:**
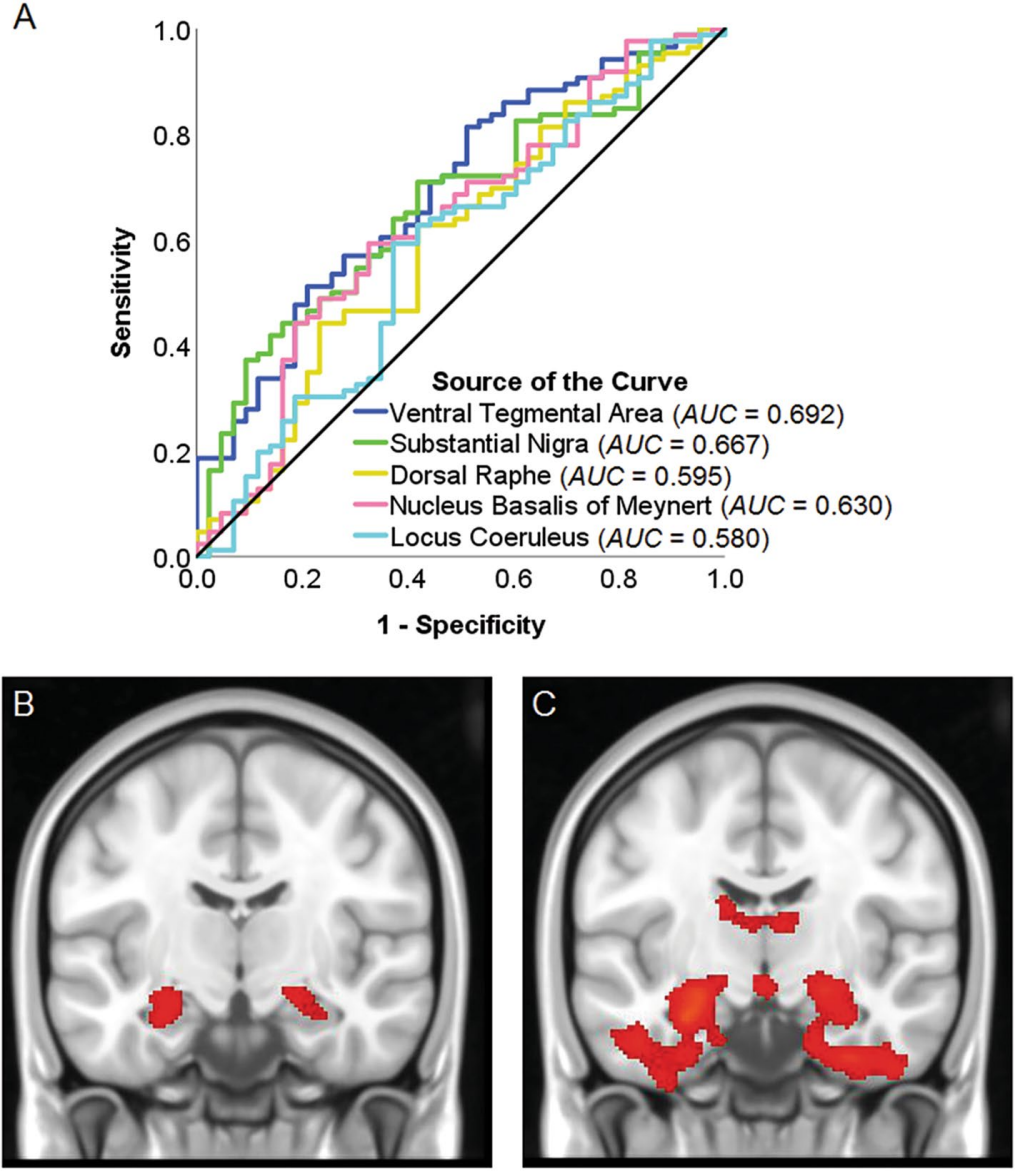
(**A**) Area under the receiver-operating characteristic curve quantifying how well Timepoint-1 monoaminergic nuclei’s signal predicts Timepoint-2 group membership. (**B**, **C**) Timepoint-1 and Timepoint-2 (respectively) voxel-by-voxel cluster level differences between the two groups (stable participants > declining participants).

The outcome of group comparisons at Timepont-1 and Timepoint-2 is shown in Fig. 5a-b. Participants with memory decline had reduced signal in the VTA at both timepoints (*p* = 0.002 and *p* < 0.001, respectively). No difference emerged from the analyses of the other nuclei. The area under the receiver-operating characteristic curve quantifying prediction of decline of the five Timepoint-1 signals is shown in Fig. 6a.

The outcome of the longitudinal analyses is shown in Fig. 5c. No group-by-timepoint interactions were found.

### MRI whole brain

Cluster-level volumetric differences at Timepoint-1 were limited to the hippocampus, bilaterally (stable participants > declining participant; Fig. 6b), while the difference at Timepoint-2 was much more extensive, covering large portions of the temporal, mediotemporal and limbic lobe (Fig. 6c).

### Association with AD biomarkers

No significant association was found between cerebrospinal concentration of biomarkers and the signal in any of the nuclei.

### Post-hoc: signal from three years prior to evidence of memory decline

A further MRI measurement acquired ~ 2 to 2.5 years (712–807 days) prior to Timepoint-1 was available for 17 of the 43 declining participants. The signal from all nuclei was extracted at this additional timepoint to characterise further its longitudinal progression. Paired-sample *t*-tests revealed no significant differences between these two timepoints for any of the nuclei.

### Post-hoc: follow up CDR measurements in the group of declining participants

Within the group of declining participants, additional ≥ 1 year follow up CDR measurements were available for 27/43 cases. Twelve of these showed evidence of further memory decline leading to dementia (CDR ≥ 1), while the other 15 remained stable at 0.5 at the available follow ups. To test whether differences existed between these two subgroups (suggesting, therefore, potential diagnostic divergences), independent-sample *t* tests (two-tailed) were run to compare global neurostructural properties and the signal from the five nuclei. No differences were found at either of the two timepoints (all *p* values > 0.05). The same analyses were rerun as 1 × 3 one-way *ANOVA*s including the third group of 16 participants for whom no CDR follow up was available. Again, no differences emerged (all *p* values > 0.05), indicating no evidence of aetiological inhomogeneities in the group of declining participants.

## Discussion

A body of research indicates that midbrain monoaminergic nuclei undergo significant cellular changes in AD before any significant brain amyloidosis or TAU hyperphosphorylation. To transpose and test this principle in a clinical study, we hypothesised that MRI T1W signal extracted from monoaminergic nuclei would show cross-sectional and longitudinal differences between cognitively unimpaired participants who would later develop memory decline and unimpaired participants remaining cognitively stable. All participants had been recruited as part of an initiative aimed at determining the multi-domain characteristics of the AD spectrum. The results indicate that signal differences in the VTA are already visible at the preclinical stage, before the onset of any memory symptom.

Although cerebrospinal fluid levels of biomarkers were not available for the totality of our sample, the partial sample with biomarkers indicates that, at Timepoint-1, declining participants already showed lower levels of cerebrospinal fluid beta amyloid compared to stable participants.

The two groups showed no differences in the DR or in the LC at either timepoints, but these nuclei showed a trend of longitudinal signal reduction among declining participants (*p* = 0.082 and *p* = 0.1, respectively). Although non significant, these trends are particularly informative for the study of memory decline. In fact, the hippocampus (the region most centrally responsible for episodic memory processing), receives multiple innervations from monoaminergic areas, including axonal projections from the VTA^33^, LC^34^, and DR^35^. In this context, our findings are consistent with results obtained in murine models^5,6^, which highlight the role of early dopaminergic changes as mechanistically involved in hippocampal and memory decline.

The definition of memory decline was based on the increase on the CDR global score (strongly dependent on the CDR memory sub-score). The CDR score is only one of five inclusion criteria for a diagnosis of mild cognitive impairment in ADNI (procedure manuals can be consulted at adni.loni.usc.edu). As a consequence, we cannot equate the CDR increase found in the group of declining participants to a proper progression to mild cognitive impairment. A diagnosis of mild cognitive impairment in ADNI can be made, among others, only in the presence of abnormal performance on the Logical Memory II subscale (from the Wechsler Memory Scale – Revised). This criterion, however, is profoundly dependent on levels of cognitive reserve and needs to be corrected based on levels of education as per procedural guidelines. As a consequence, rather than studying a form of multi-componential decline, we chose to focus on the sole functional decline captured by longitudinal and non-fluctuating CDR increase. In addition, although indirectly important at an interpretational level to help characterise the early stages of AD, these findings do not directly transfer to “preclinical AD”, a concept that is based on biological criteria^36^. Longitudinal biomarker data were available only for a proportion of datasets and this prevented us from testing any AD-centred hypothesis in a direct way. The use of the CDR, however, allows one to capture memory difficulties outside of the clinical setting with the additional benefit of the help of an informant. There is evidence, in fact, that individuals may have sufficient resources to sustain a “well-defined” problem such as those typical of laboratory settings, but these may not be sufficient to address “ill-defined” real-world problems^37^. In the search for early AD biomarkers it is paramount to investigate the contribution that an instrument susceptible to memory changes of minimal intensity such as the CDR may offer.

We also investigated the differences between the two groups of participants by analysing their brain with standard voxel-based morphometry. The results revealed significant differences at both timepoints in associative areas known to be affected in AD, with the hippocampus being the epicentre of the detected differences at Timepoint-1.

This study is not exempt from limitations. First, biomarker data were not bound to the longitudinal MRI timescale, and for this reason we included a single measurement obtained in proximity (< 1 year) of Timepoint-1, with no measurement for Timepoint-2. Second, the methodology was applied retrospectively to MRI images that had not been explicitly collected to highlight small subcortical nuclei. There are other types of anatomical MRI images that can be used to quantify T1W signal from these regions, such as fast low-angle shot sequences^38^. Monoaminergic nuclei are very small and particular care has to be taken when MRI images are used to characterise these regions. For this reason, we ascertained test–retest signal reliability and we adopted a longitudinal design for statistical inference. By doing so, we minimised the chances that the findings could be influenced by artefactual signal variability. Other MRI specifications can, however, be implemented in the future for a more detailed characterisation of the target regions.

In conclusion, we found evidence in support of a statistically predictive link between the VTA and memory decline in a group of cognitively unimpaired participants enrolled in an AD-centred initiative. This confirms and extends previous findings emerged with a cross-sectional design in a cohort of healthy adults^10^, and supports the findings of other neuroimaging studies that have highlighted a role of damage in this nucleus in patients with MCI^39^. Dopaminergic neurotransmission may contribute to the pathogenesis of AD^40,41^, and further longitudinal investigations carried out in asymptomatic individuals are warranted to characterise the preclinical stage of AD more in detail and thus help devise tailored interventions to be administered at an established preclinical stage. Indeed, there is a large consensus that effective treatment of Alzheimer’s disease can only be achieved by a thorough understanding of its causal mechanisms, and that any treatment to be effective has to be started as early as possible. More importantly, it has been suggested that the most effective treatment strategy might be one that is aimed at multiple pathological processes as it is already the case for other chronic diseases^42^. The findings reported in this study contribute to this goal by providing evidence in support of a potential additional early therapeutic target. Although early studies of seligiline in patients with established AD dementia have reported no clinical benefit^43^, other monoamine oxidase inhibitors^44,45^ and dopamine agonists^46^ seem to induce more promising effects, and limited evidence exists in favour of a positive effect of a dopamine agonist in mild cognitive impairment as well^47^, warranting a deeper focus on this therapeutic avenue.

## Supplementary information

Supplementary file1
