# Supplementary material for: Reduced monoaminergic nuclei MRI signal detectable in pre-symptomatic older adults with future memory decline
Source: Sci Rep. 2020 Oct 30;10:18707. doi: 10.1038/s41598-020-71368-1 (PMC7603335; doi:10.1038/s41598-020-71368-1)
Supplement: Supplementary file 1 — Supplementary file1 [file 41598_2020_71368_MOESM1_ESM.docx]

**Reduced monoaminergic nuclei MRI signal detectable in pre-symptomatic older adults with future memory decline**

Annalena Venneri PhD^#^, Matteo De Marco PhD^#^,

for the Alzheimer’s Disease Neuroimaging Initiative*

Department of Neuroscience, University of Sheffield, Sheffield, UK

**Supplementary Material**

**Analysis of Participants with Fluctuating CDR**

An additional group of 35 participants was included in a subsidiary section of the study. These were individuals recruited in ADNI-2 as normal controls (thus having a CDR = 0), who then scored 0.5 at one of the later study phases, reverting to 0 at one of the subsequent measurements. As with the group of declining participants included in the main body of the study, Timepoint-1 was defined as the last measurement with a CDR = 0 before conversion and Timepoint-2 corresponded to the CDR = 0.5 phase. Demographic, biomarker and global neurostructural indices of this group (in relation to the other two study groups) are reported in **Supplementary Table 1**.

The same methodology described in the Section 2 of the main article was applied to the processing of MRI data for the extraction of the T1W signal from each of the five nuclei and from the 12 white-matter regions. Data analysis was thus rerun taking into account the inclusion of a further group. One-way *ANOVA*s were run to test the effect of the variable ‘group’ at Timepoint-1 and Timepoint-2, followed by Šidák-corrected *post hoc* comparisons. Likewise, five 3×2 group-by-timepoint mixed *ANOVA* models were run to test for the presence of difference in the longitudinal shift from Timepoint-1 to Timepoint-2.

The results of these analyses are shown in **Supplementary Fig. 1**. The only models where a significant effect of ‘group’ was found were Timepoint-1 and Timepoint-2 analyses of VTA signal. *Post hoc* comparisons revealed that the only significant between-group differences were those between stable and declining participants, replicating the findings of the main analyses. VTA signal of fluctuating participants was not different from that of the other two groups.

The procedures to define membership in the group of declining participants were based on objective evidence of non-fluctuating CDR decline. Although the entire list of timepoints was inspected to confirm that decline was not characterised by fluctuations (including all available follow ups), we cannot rule out possible fluctuations gone undetected (e.g., reversions to CDR = 0 after dropout, or, considering the rather advanced age of participants, a more general impossibility of following them up to confirm possible CDR reversions). At the same time, it is also possible that a number of fluctuating participants may have been, in fact, patients at an early disease stage who may have simply shown a temporary reversion due to beneficial lifestyle changes introduced after receiving a diagnosis of functional decline based on a change in their CDR score. In summary, although the separation of declining and fluctuating participants was based on objective evidence, there is a theoretical rationale by which a degree of continuity can be defined between the two groups. The results are consistent with this view, and indicate that the group-level distribution of monoaminergic signal in fluctuating adults fell in between stable and declining adults, without showing any significant difference with these two groups.

**Supplementary Table 1.** Demographic and global neurostructural indices of fluctuating participants

| **Variable** | | **Fluctuating Participants (*n* = 35)** | | **Sig. *ANOVA* test** |
| --- | --- | --- | --- | --- |
| *Demographic indices* | | | | |
| Age _(years)_ | | 75.11 (7.54) | | 0.016 ^a^ |
| Education _(years)_ | | 16.71 (2.56) | | 0.395 |
| Gender _(f/m)_ | | 21/14 | | 0.954 |
| ApoE genotype _(ε2ε2/ε2ε3/ε2ε4/ε3ε3/ε4ε3/ε4ε4)_ | | 0/7/0/19/8/1 | | 0.431 |
| Timepoints-1–2 distance _(days_) | | 363.80 (245.50) | | 0.021 ^b,d^ |
| Total intracranial volume (ml) | | 1473.83 (121.15) | | 0.857 |
| *Cerebrospinal fluid AD biomarkers* | | | | |
| Beta Amyloid_1-42_ (pg/ml) | | 1364.50 (595.04) | | 0.037 ^c^ |
| Total TAU (pg/ml) | | 244.18 (97.13) | | 0.347 |
| Phosphorylated TAU (pg/ml) | | 22.33 (9.99) | | 0.178 |
| *Global neurostructural indices – Timepoint-1* | | | | |
| Grey matter volume (ml) | | 607.27 (59.72) | | 0.006 ^c^ |
| White matter volume (ml) | | 408.42 (44.96) | | 0.119 |
| Left hippocampal volume (ml) | | 2.50 (0.35) | | 0.001 ^c^ |
| Right hippocampal volume (ml) | 2.59 (0.36) | | < 0.001 ^c^ | |
| *Global neurostructural indices – Timepoint-2* | | | | |
| Grey matter volume (ml) | 597.79 (58.75) | | 0.003 ^c^ | |
| White matter volume (ml) | 402.95 (48.25) | | 0.179 | |
| Left hippocampal volume (ml) | 2.47 (0.35) | | < 0.001 ^c^ | |
| Right hippocampal volume (ml) | 2.56 (0.37) | | < 0.001 ^c^ | |

Cerebrospinal fluid analyses were run on 131 out of 164 participants; ^a^: Declining > Stable, ^b^: Stable > Fluctuating, ^c^: Stable > Declining, ^d^: Declining > Fluctuating(see Table 1 in main manuscript for values of stable and declining participants)

**Supplementary Fig. 1**


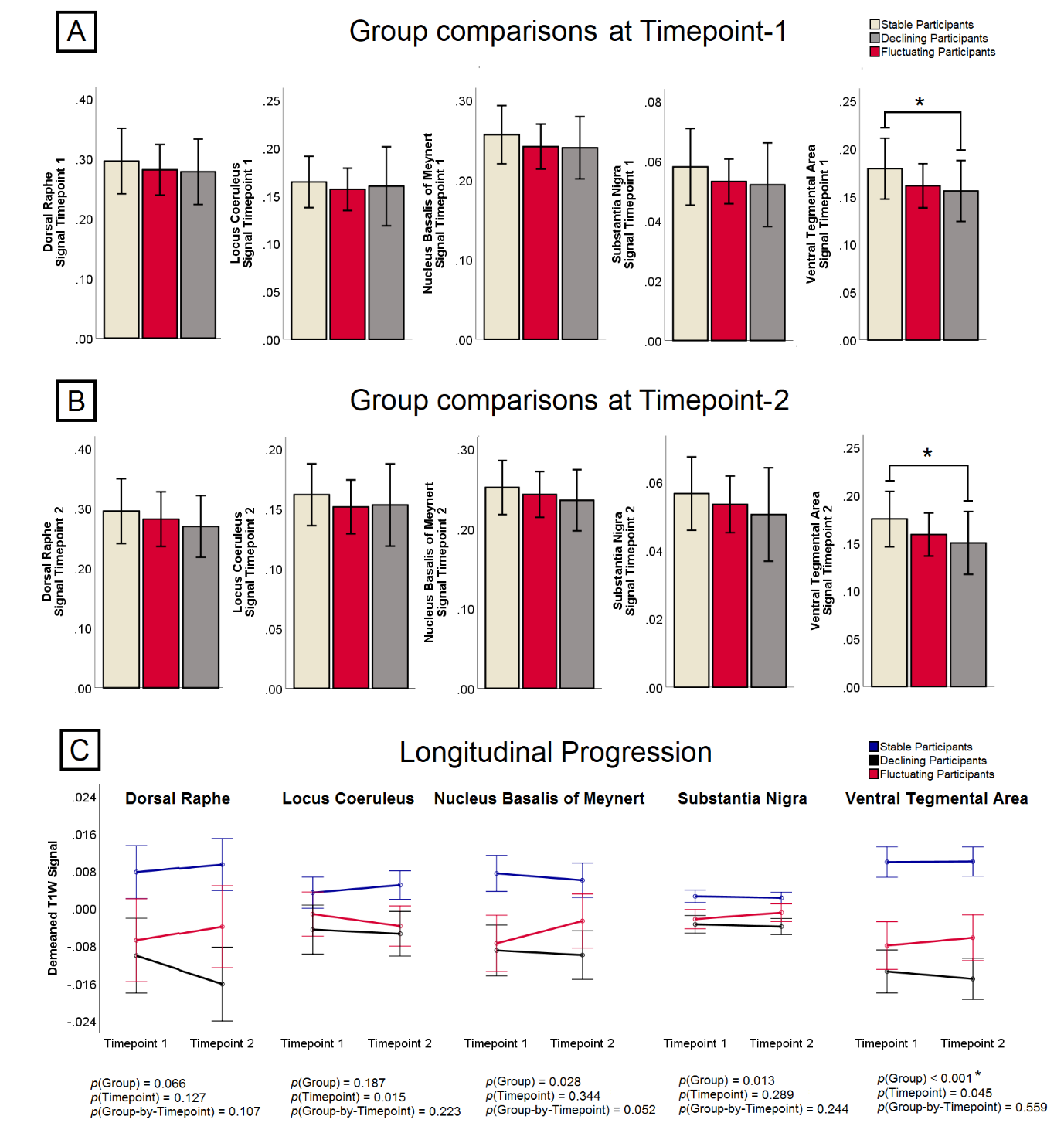


(A-B): Between-sample comparisons across the three groups: stable (*n* = 86), declining (*n* = 43) and fluctuating (*n* = 35) participants at the two timepoints. Graphs indicate arithmetical means and error bars represent one standard deviation. (C): Graphical representation of the mixed *ANOVA* models. Error bars indicate the standard error of the mean. *: *p* < 0.01.
